# Group-Based Smoking Cessation Program for Older Adults with Serious Mental Illness

**DOI:** 10.1093/geroni/igab046.3131

**Published:** 2021-12-17

**Authors:** Heather Leutwyler, Erin Hubbard

**Affiliations:** 1 University of California, San Francisco, San Francisco, California, United States; 2 UCSF, UCSF, California, United States

## Abstract

Smoking is one of the most important modifiable risk factors for excess morbidity and mortality in older adults with serious mental illness (SMI). Many older smokers with SMI are reportedly motivated to quit, however evidence-based treatment targeting this vulnerable group is limited. To address an urgent need to identify interventions that assist smoking cessation efforts, we are conducting a pilot two-arm randomized controlled trial (RCT) targeting adults with SMI. Our VIdeogame-based Physical activity (“VIP”) smoking cessation intervention includes: a) group videogame-based physical activity intervention (50 minutes, 3X/week for 12 weeks), b) pharmacotherapy (bupropion or nicotine replacement therapy), and c) smoking cessation counseling. Upon completion of the 12 week program, participants in the VIP and control groups completed a semi-structured interview in order to determine how the program impacted their smoking cessation. To date, six participants completed an interview. Participants described how the program helped with smoking cessation because it allowed them to “face their addiction” and learn more about why they smoke and how to quit. The program provided the structure, resources, and encouragement needed to start the process of quitting. Finally, they enjoyed having the game time as a distraction from smoking. Older adults with SMI need support, resources, and group-based exercise as they begin quitting and practice the skills needed to quit.

